# Supplementary material for: Core–Shell Polyvinyl Alcohol (PVA) Base Electrospinning Microfibers for Drug Delivery
Source: Polymers (Basel). 2023 Mar 21;15(6):1554. doi: 10.3390/polym15061554 (PMC10056133; doi:10.3390/polym15061554)
Supplement: Supplementary file 1 [file polymers-15-01554-s001.zip › polymers-2268391-supplementary.pdf]

polymers

MDPI

Supplementary Material

U-ES microfibers of PVA 21% as a function of flow rate and voltage

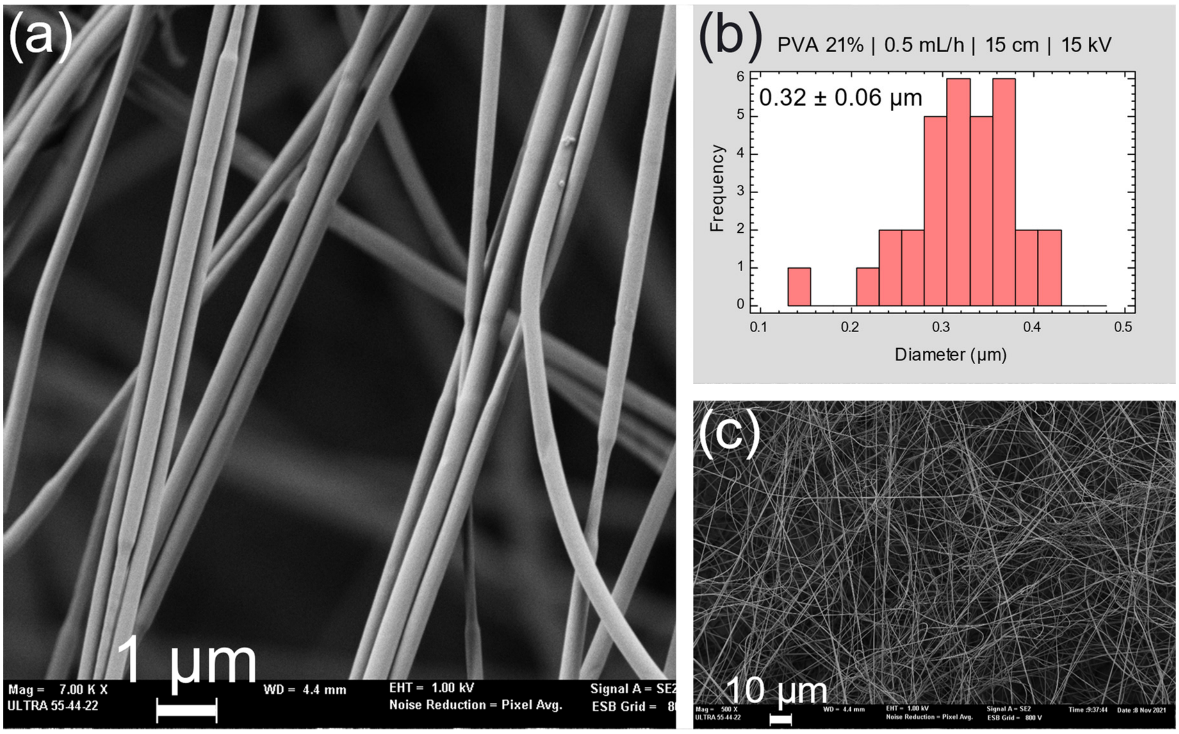

Figure S1. Microfibers of PVA 21% manufactured with flow 0.5 mL/h at 15 cm and 15 kV by FESEM (a, c) and histogram diameter results (mean ± SD = 0.32 ± 0.06 µm; range from 0.149 to 0.427) (b).

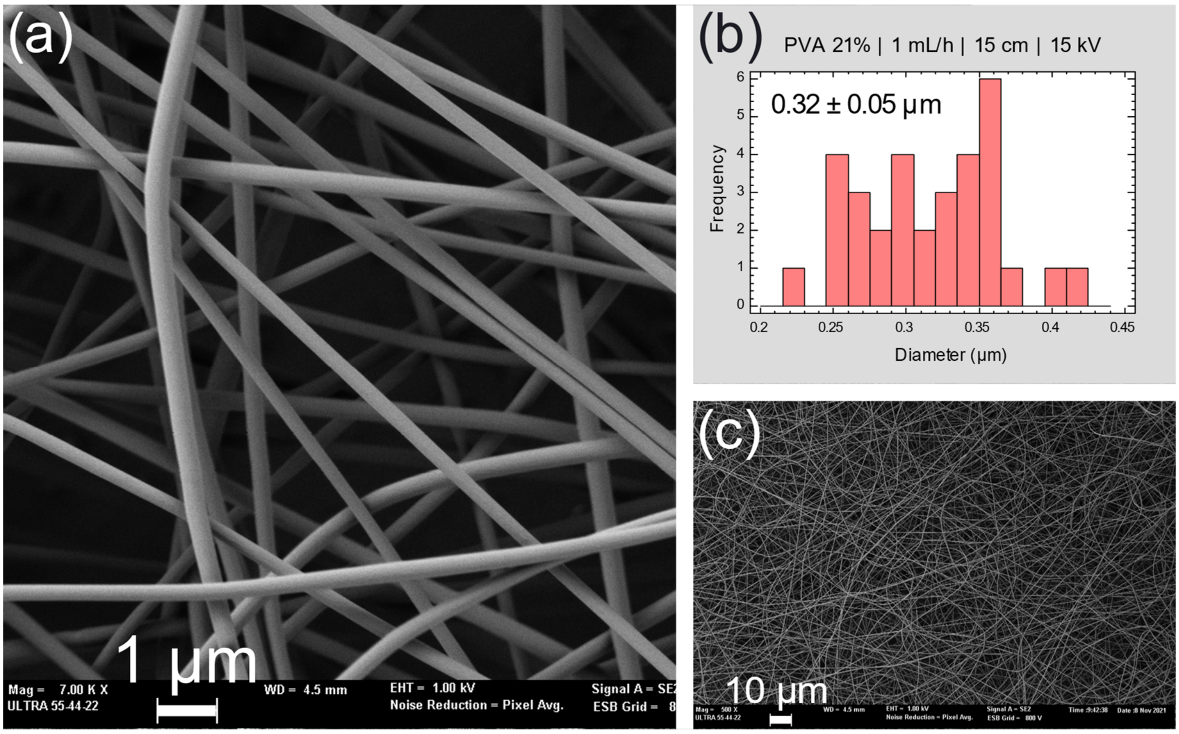

Figure S1. Microfibers of PVA 21% manufactured with flow 1 mL/h at 15 cm and 15 kV by FESEM (a, c) and histogram diameter results (mean ± SD = 0.32 ± 0.05 µm; range from 0.217 to 0.415) (b).

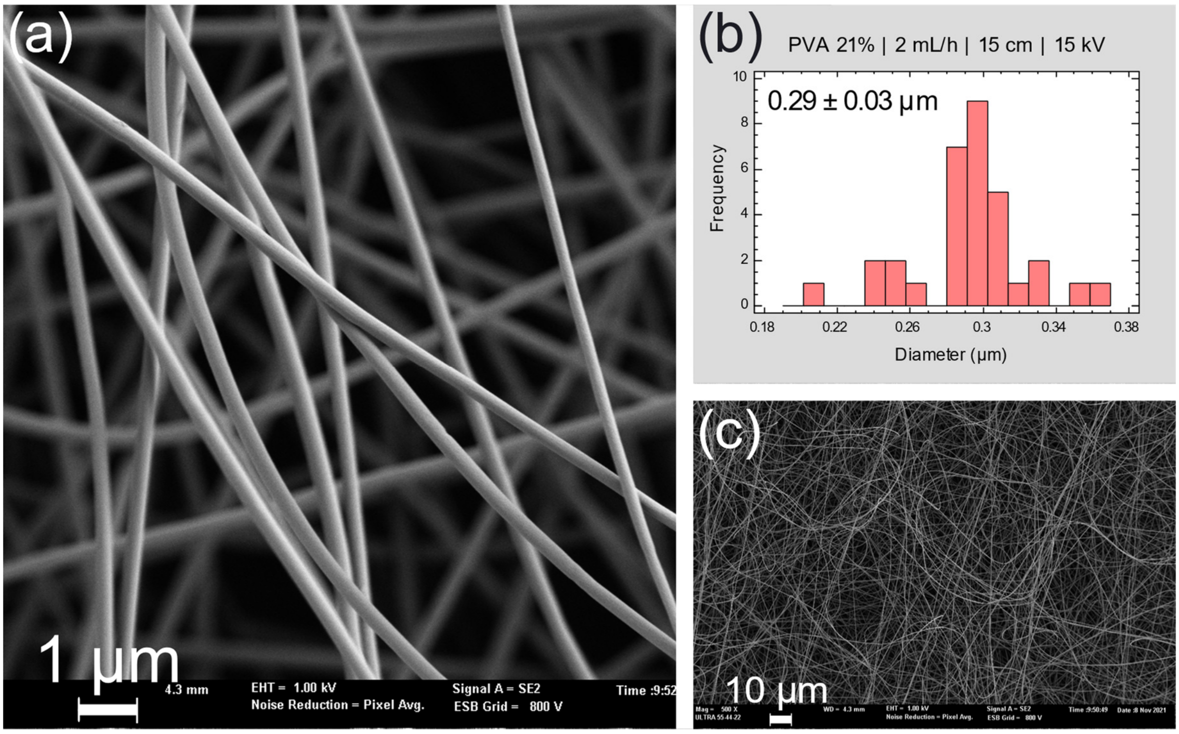

Figure S3. Microfibers of PVA 21% manufactured with flow 2 mL/h at 15 cm and 15 kV by FESEM (a, c) and histogram diameter results (mean ± SD = 0.29 ± 0.03 µm; range from 0.203 to 0.361) (b).

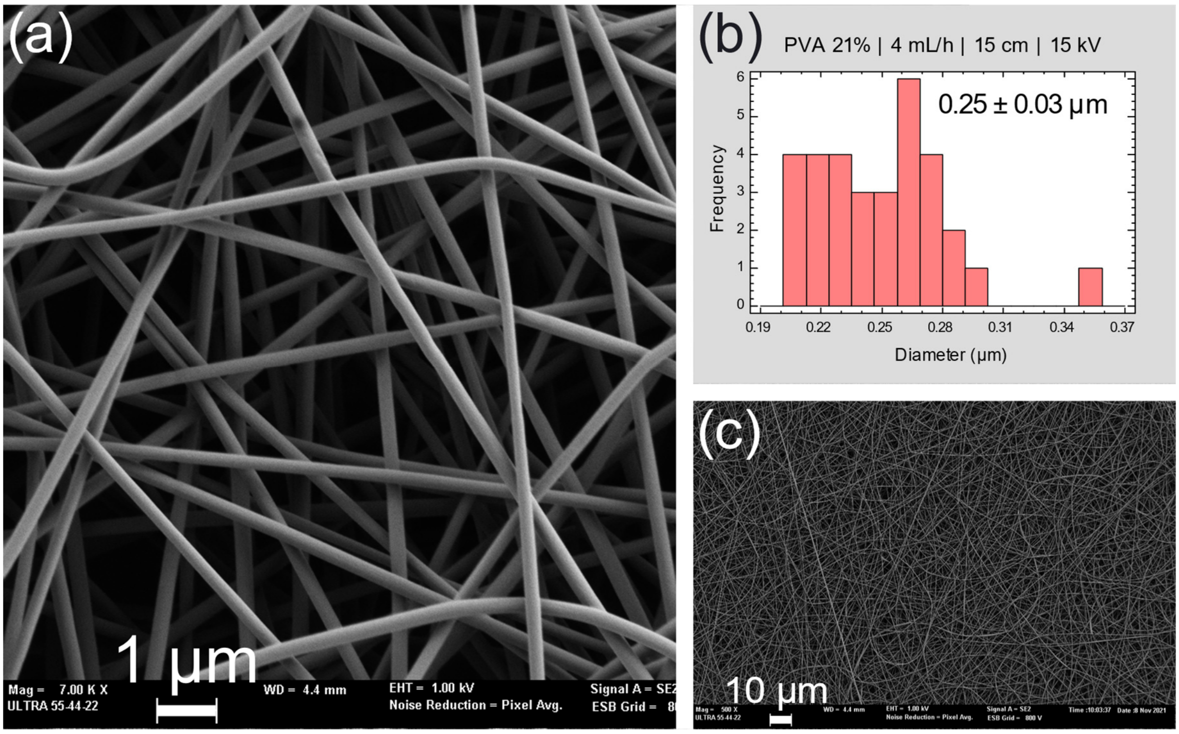

Figure S4. Microfibers of PVA 21% manufactured with flow 4 mL/h at 15 cm and 15 kV by FESEM (a, c) and histogram diameter results (mean ± SD = 0.25 ± 0.03 µm; range from 0.206 to 0.357) (b).

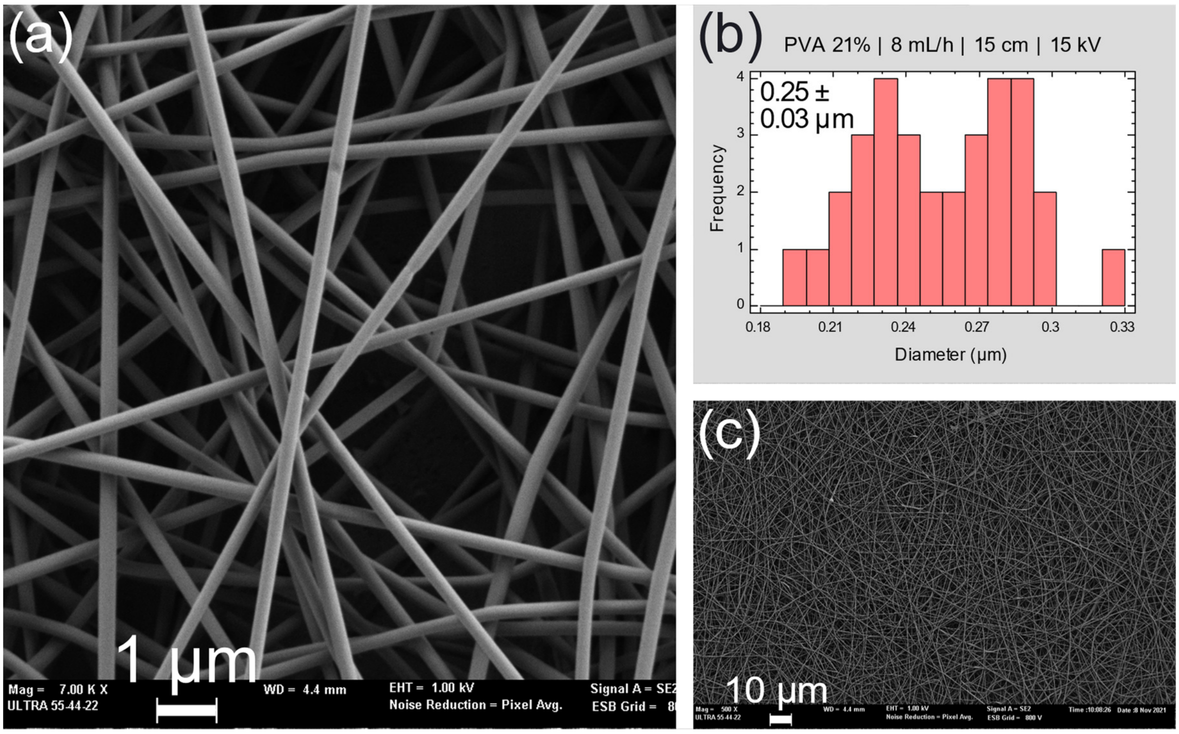

Figure S5. Microfibers of PVA 21% manufactured with flow 8 mL/h at 15 cm and 15 kV by FESEM (a, c) and histogram diameter results (mean ± SD = 0.26 ± 0.03 µm; range from 0.194 to 0.322) (b).

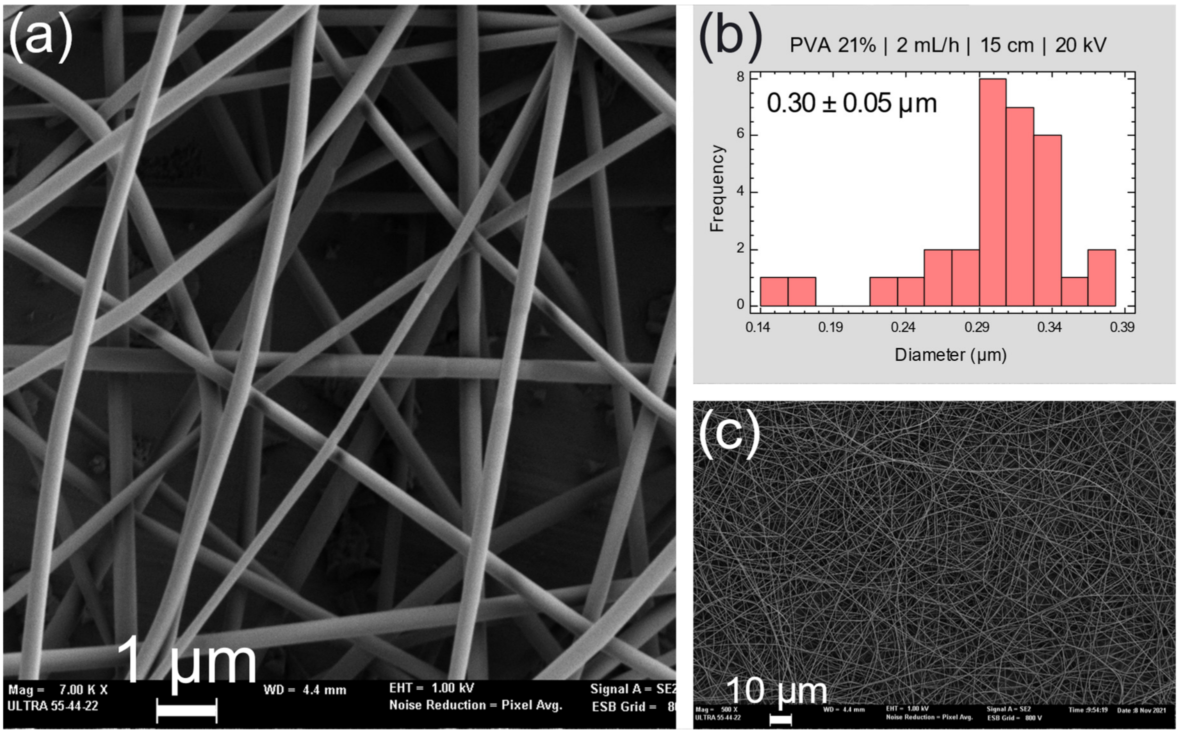

Figure S6. Microfibers of PVA 21% manufactured with flow 2 mL/h at 15 cm and 20 kV by FESEM (a, c) and histogram diameter results (mean ± SD = 0.30 ± 0.05 µm; range from 0.155 to 0.38) (b).

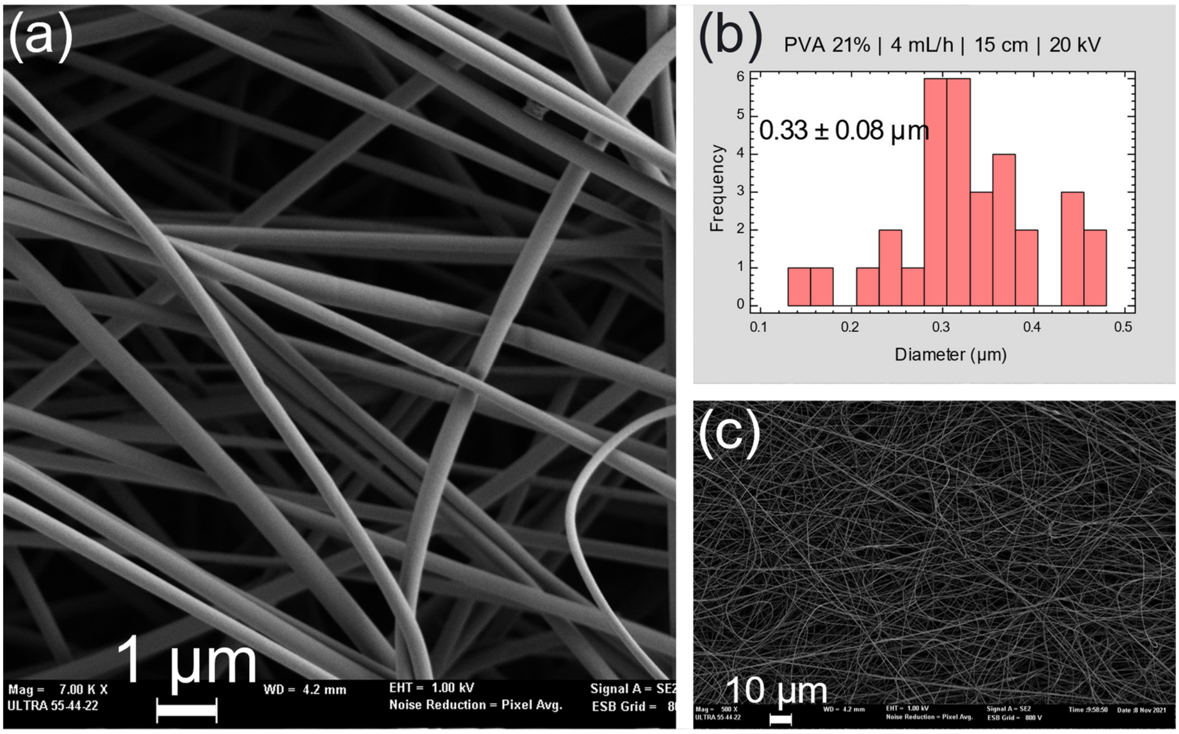

Figure S7. Microfibers of PVA 21% manufactured with flow 4 mL/h at 15 cm and 20 kV by FESEM (a, c) and histogram diameter results (mean ± SD = 0.33 ± 0.08 µm; range from 0.152 to 0.464) (b).

C-ES microfibers of PLLA 6% 80:20 with core of mQ water as a function of inner flow rate

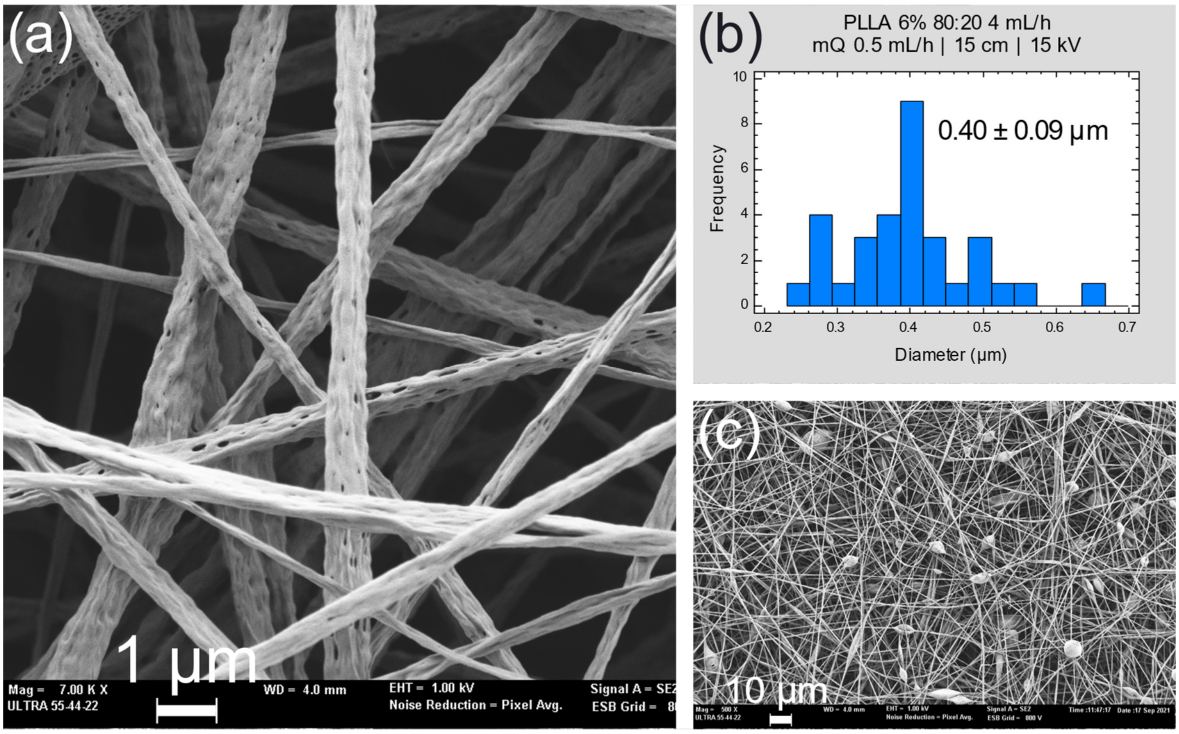

Figure S8. Coaxial microfibers of PLLA 6% chloroform:DMF 80:20 shell and mQ water core manufactured with shell - core flow 4 - 0.5 mL/h at 15 cm and 15 kV by FESEM (a, c) and histogram diameter results (mean ± SD = 0.40 ± 0.09 µm; range from 0.255 to 0.664) (b).

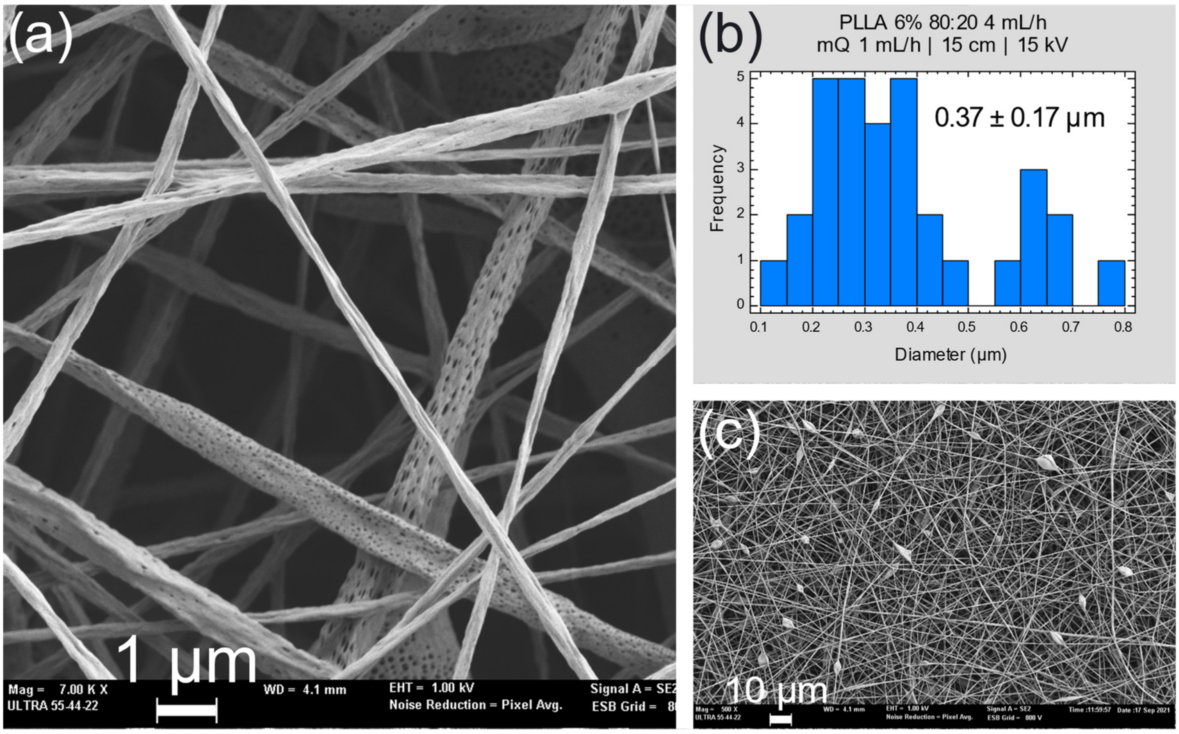

Figure S9. Coaxial microfibers of PLLA 6% chloroform:DMF 80:20 shell and mQ water core manufactured with shell – core flow 4 – 1 mL/h at 15 cm and 15 kV by FESEM (a, c) and histogram diameter results (mean ± SD = 0.37 ± 0.17 µm; range from 0.137 to 0.758) (b).

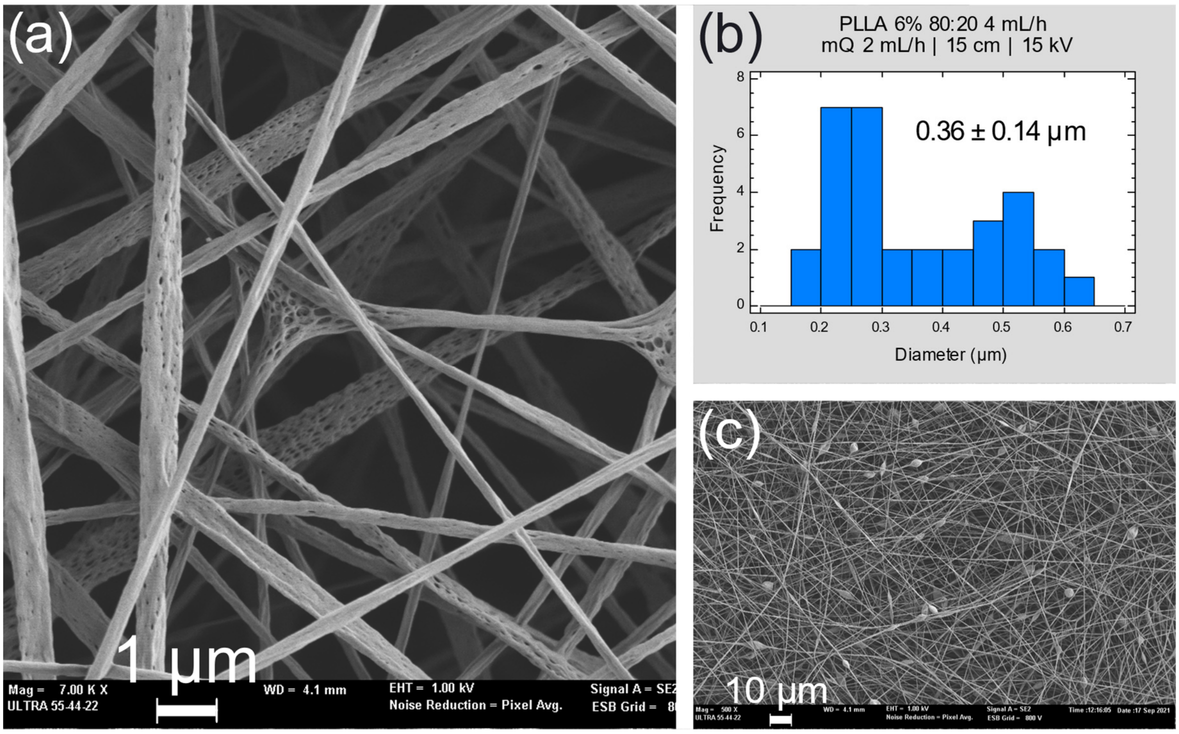

Figure S10. Coaxial microfibers of PLLA 6% chloroform:DMF 80:20 shell and mQ water core manufactured with shell - core flow 4 - 2 mL/h at 15 cm and 15 kV by FESEM (a, c) and histogram diameter results (mean ± SD = 0.36 ± 0.14 µm; range from 0.171 to 0.63) (b).

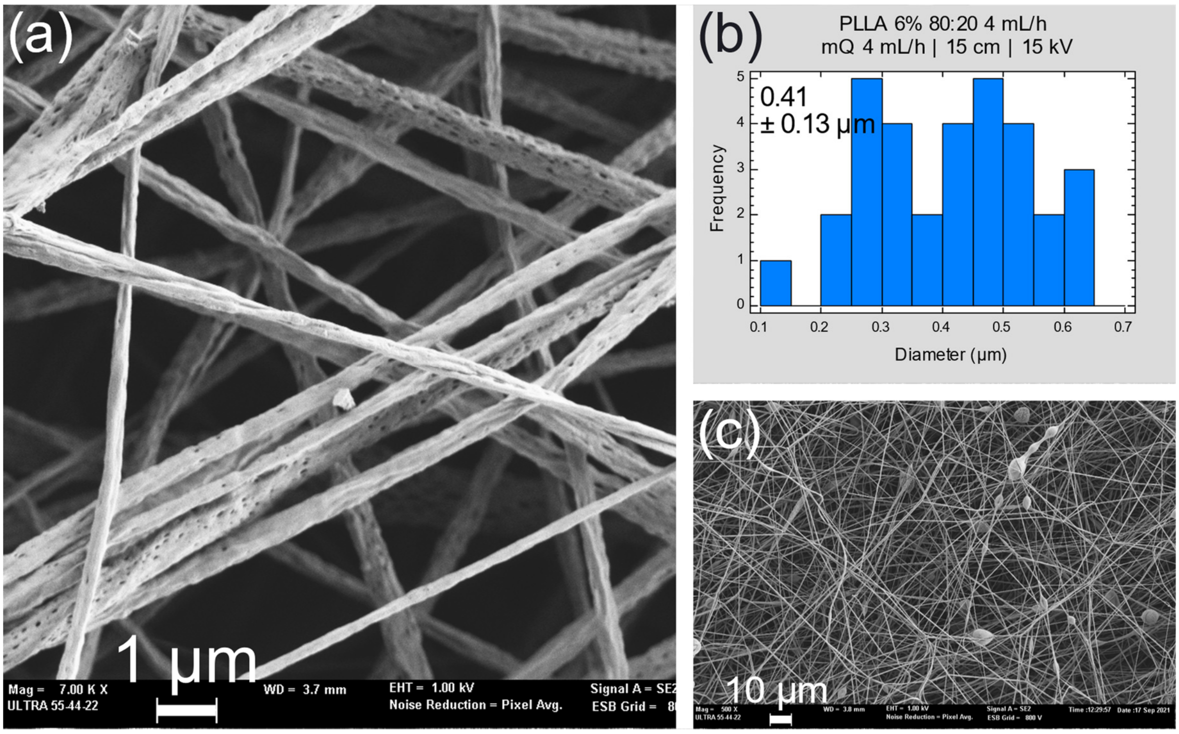

Figure S11. Coaxial microfibers of PLLA 6% chloroform:DMF 80:20 shell and mQ water core manufactured with shell – core same flow 4 - 4 mL/h at 15 cm and 15 kV by FESEM (a, c) and histogram diameter results (mean ± SD = 0.41 ± 0.13 µm; range from 0.149 to 0.647) (b).

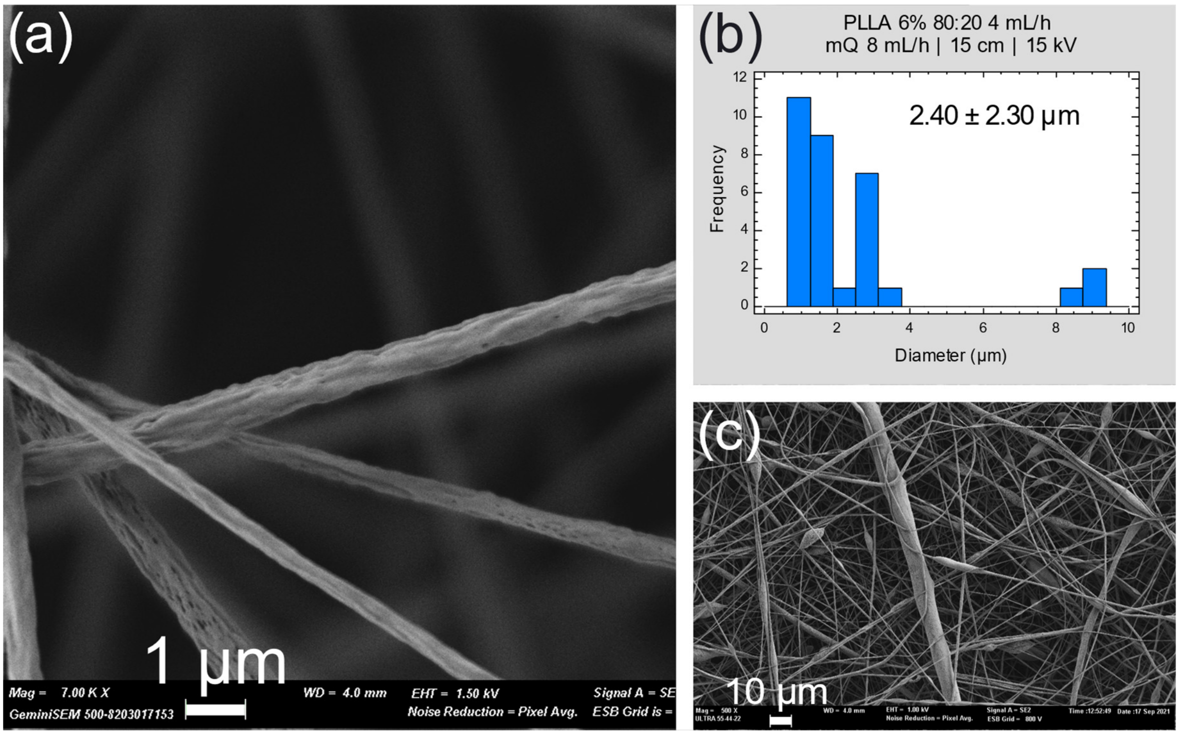

Figure S12. Coaxial microfibers of PLLA 6% chloroform:DMF 80:20 shell and mQ water core manufactured with shell – core flow 4 - 8 mL/h at 15 cm and 15 kV by FESEM (a, c) and histogram diameter results (mean ± SD = 2.4 ± 2.3 µm; range from 0.684 to 9.213) (b).

C-ES microfibers of PLLA 6% 80:20 | PVA 8% as a function of inner flow rate and voltage

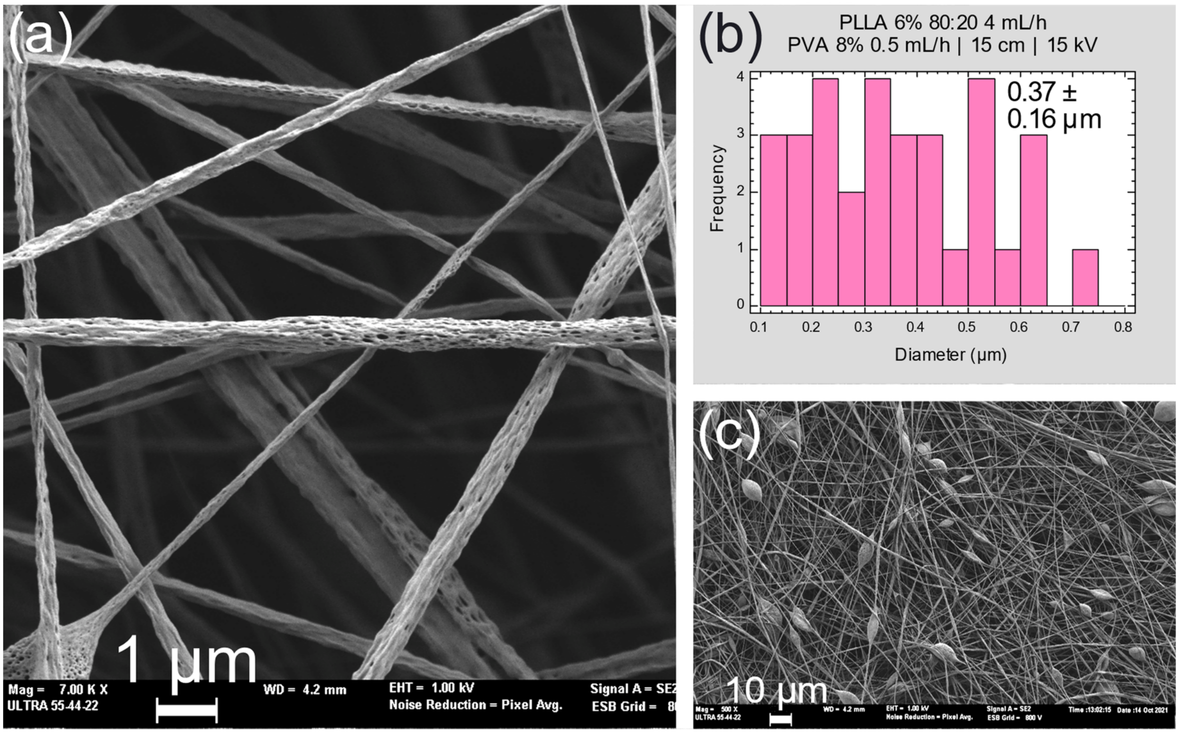

Figure S13. Coaxial microfibers of PLLA 6% chloroform:DMF 80:20 shell and PVA 8% core manufactured with shell – core flow 4 – 0.5 mL/h at 15 cm and 15 kV by FESEM (a, c) and histogram diameter results (mean ± SD = 0.37 ± 0.16 µm; range from 0.128 to 0.732) (b).

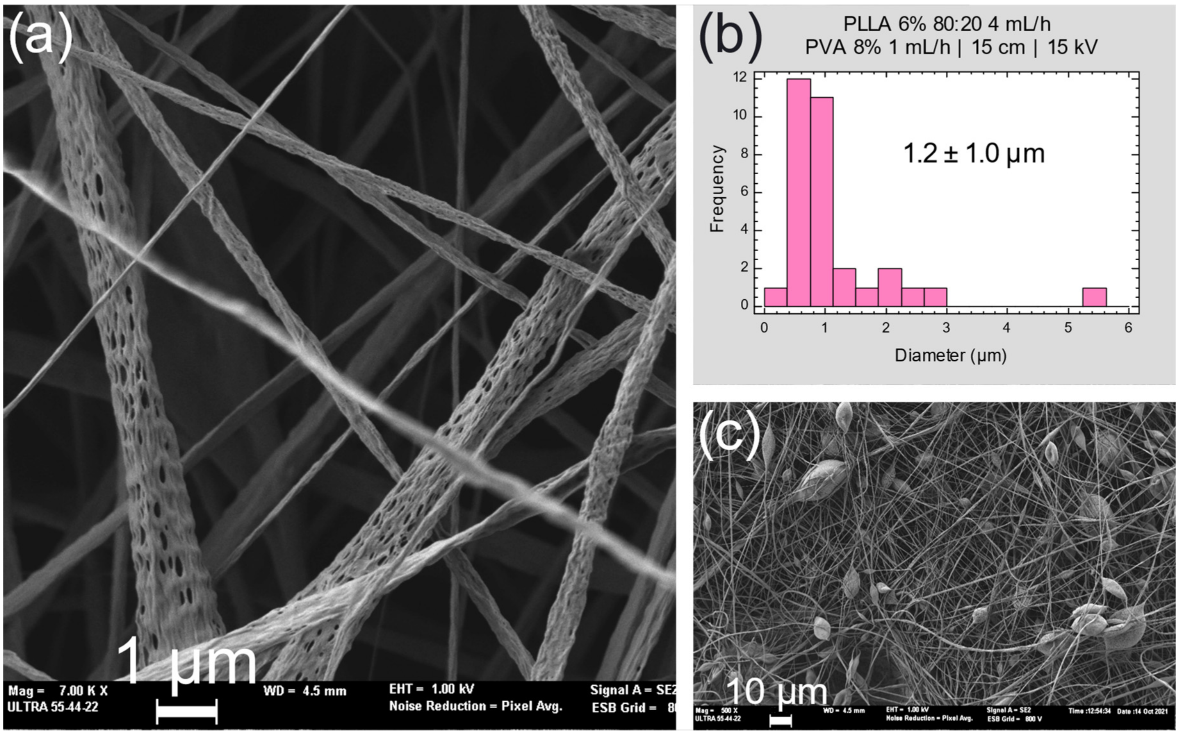

Figure S14. Coaxial microfibers of PLLA 6% chloroform:DMF 80:20 shell and PVA 8% core manufactured with shell – core flow 4 – 1 mL/h at 15 cm and 15 kV by FESEM (a, c) and histogram diameter results (mean ± SD = 1.2 ± 1.0 µm; range from 0.356 to 5.517) (b).

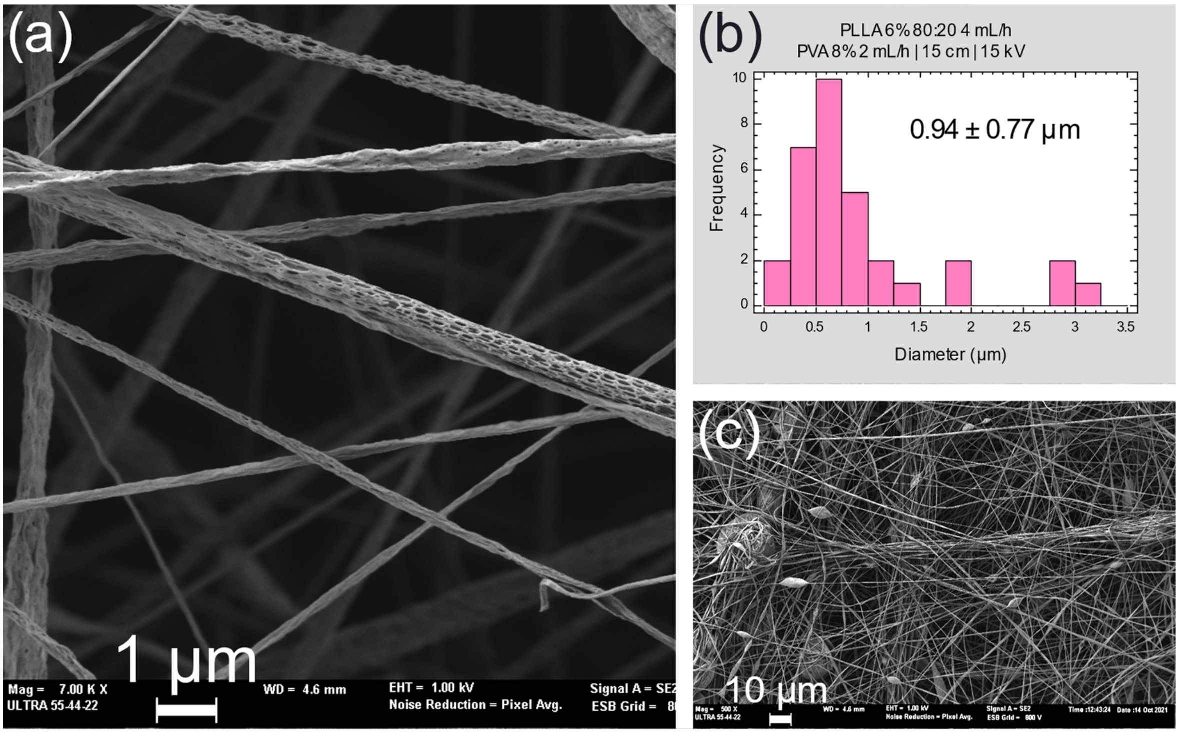

Figure S15. Coaxial microfibers of PLLA 6% chloroform:DMF 80:20 shell and PVA 8% core manufactured with shell – core flow 4 – 2 mL/h at 15 cm and 15 kV by FESEM (a, c) and histogram diameter results (mean ± SD = 0.94 ± 0.77 µm; range from 0.229 to 3.174) (b).

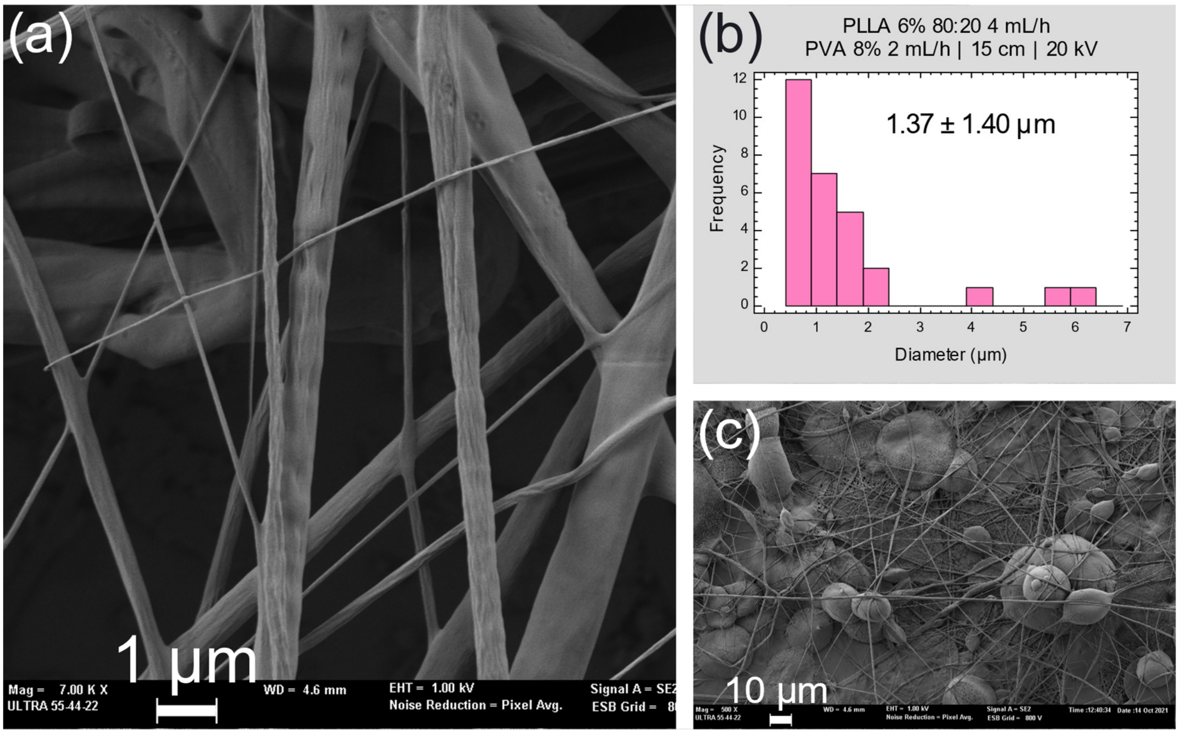

Figure S16. Coaxial microfibers of PLLA 6% chloroform:DMF 80:20 shell and PVA 8% core manufactured with shell – core flow 4 – 2 mL/h at 15 cm and 20 kV by FESEM (a, c) and histogram diameter results (mean ± SD = 1.37 ± 1.4 µm; range from 0.243 a 6.108) (b).

C-ES microfibers of PLLA 9% 80:20 | PVA 8% as a function of flow rate and voltage

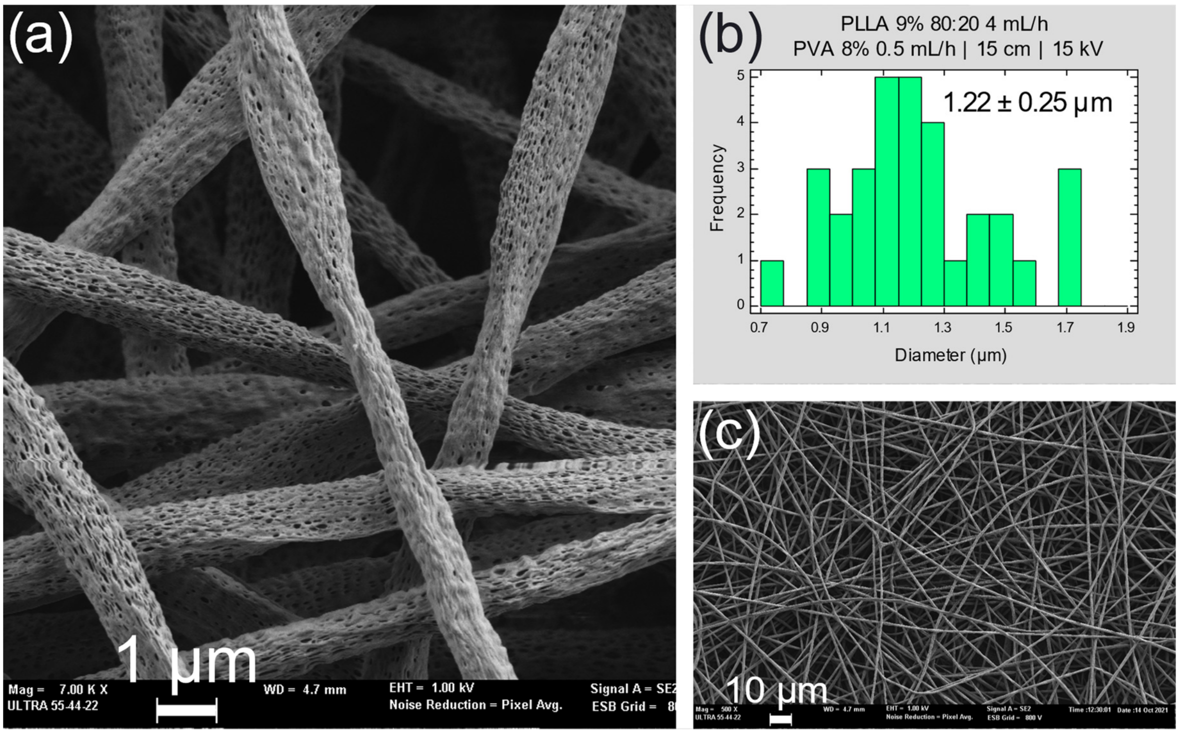

Figure S17. Coaxial microfibers of PLLA 9% chloroform:DMF 80:20 shell and PVA 8% core manufactured with shell – core flow 4 – 0.5 mL/h at 15 cm and 15 kV by FESEM (a, c) and histogram diameter results (mean ± SD = 1.22 ± 0.25 μm; range from 0.75 to 1.736) (b).

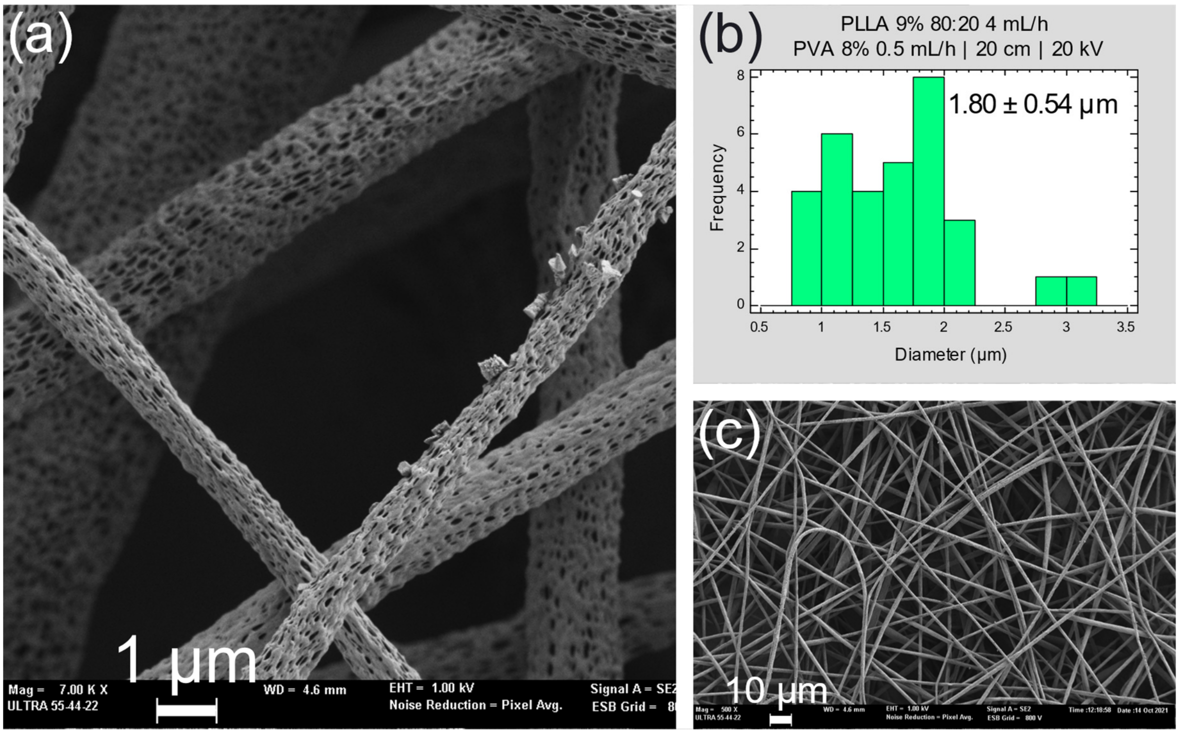

Figure S18. Coaxial microfibers of PLLA 9% chloroform:DMF 80:20 shell and PVA 8% core manufactured with shell – core flow 4 – 0.5 mL/h at 20 cm and 20 kV by FESEM (a, c) and histogram diameter results (mean ± SD = 1.80 ± 0.54 μm; range from 0.79 to 3.233) (b).

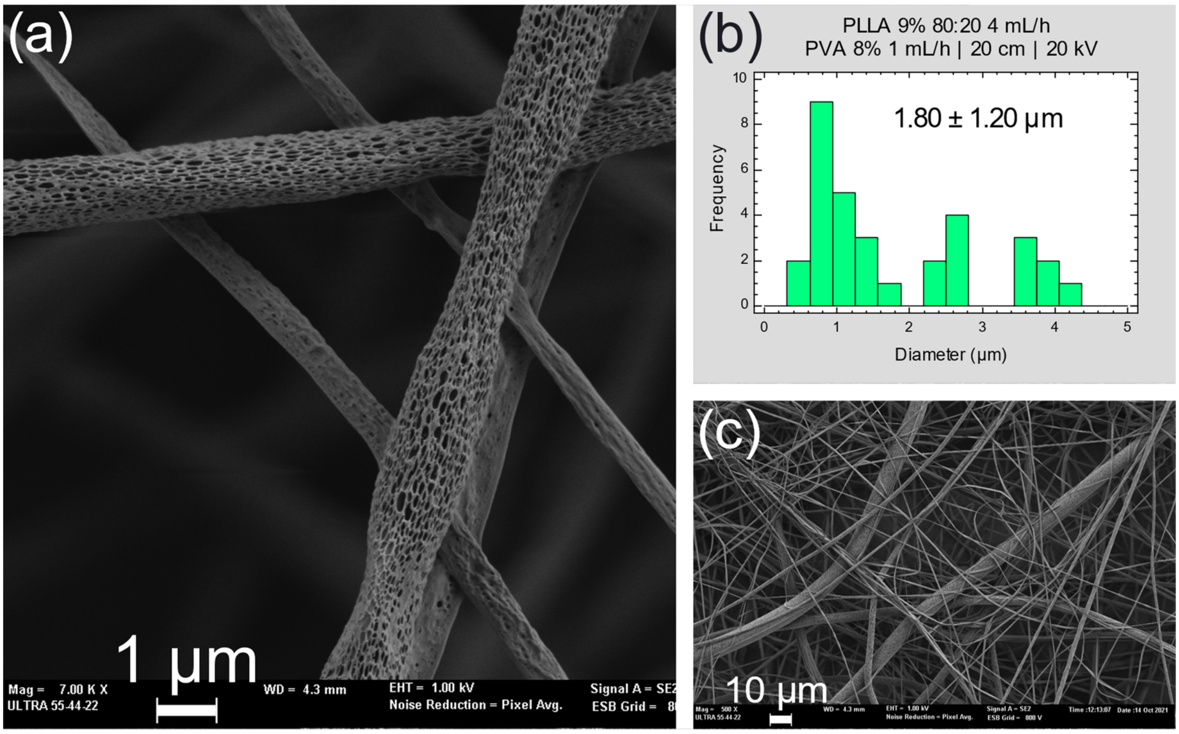

Figure S19. Coaxial microfibers of PLLA 9% chloroform:DMF 80:20 shell and PVA 8% core manufactured with shell – core flow 4 – 1 mL/h at 20 cm and 20 kV by FESEM (a, c) and histogram diameter results (mean ± SD = 1.80 ± 1.20 µm; range from 0.555 to 4.312) (b).

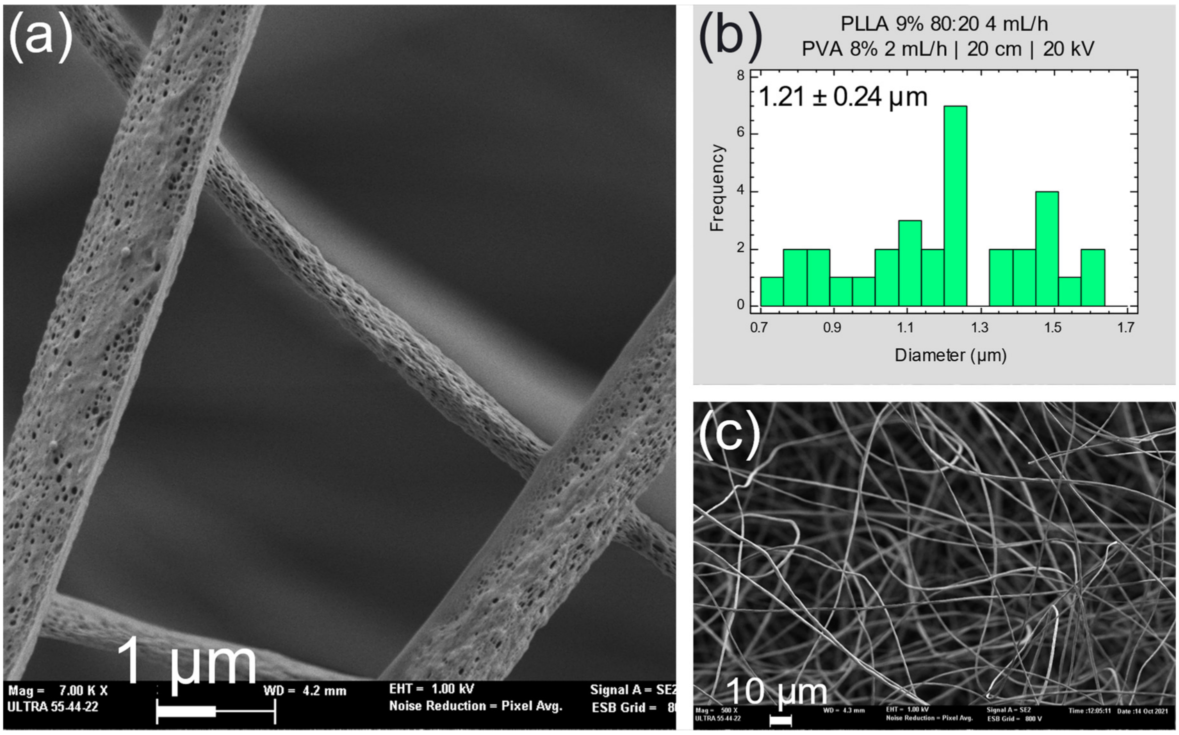

Figure S20. Coaxial microfibers of PLLA 9% chloroform:DMF 80:20 shell and PVA 8% core manufactured with shell – core flow 4 – 2 mL/h at 20 cm and 20 kV by FESEM (a, c) and histogram diameter results (mean ± SD = 1.21 ± 0.24 µm; range from 0.75 to 1.579) (b).

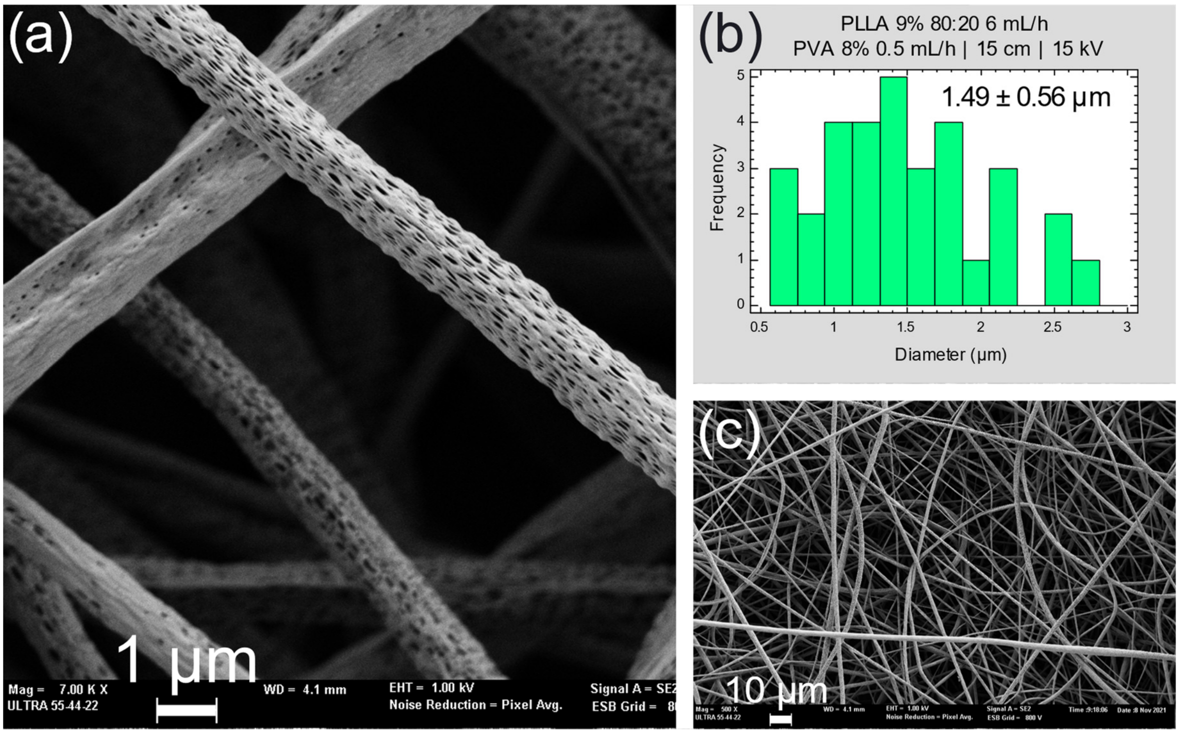

Figure S21. Coaxial microfibers of PLLA 9% chloroform:DMF 80:20 shell and PVA 8% core manufactured with shell – core flow 6 – 0.5 mL/h at 15 cm and 15 kV by FESEM (a,c) and histogram diameter results (mean ± SD = 1.49 ± 0.56 µm; range from 0.566 to 2.657) (b).

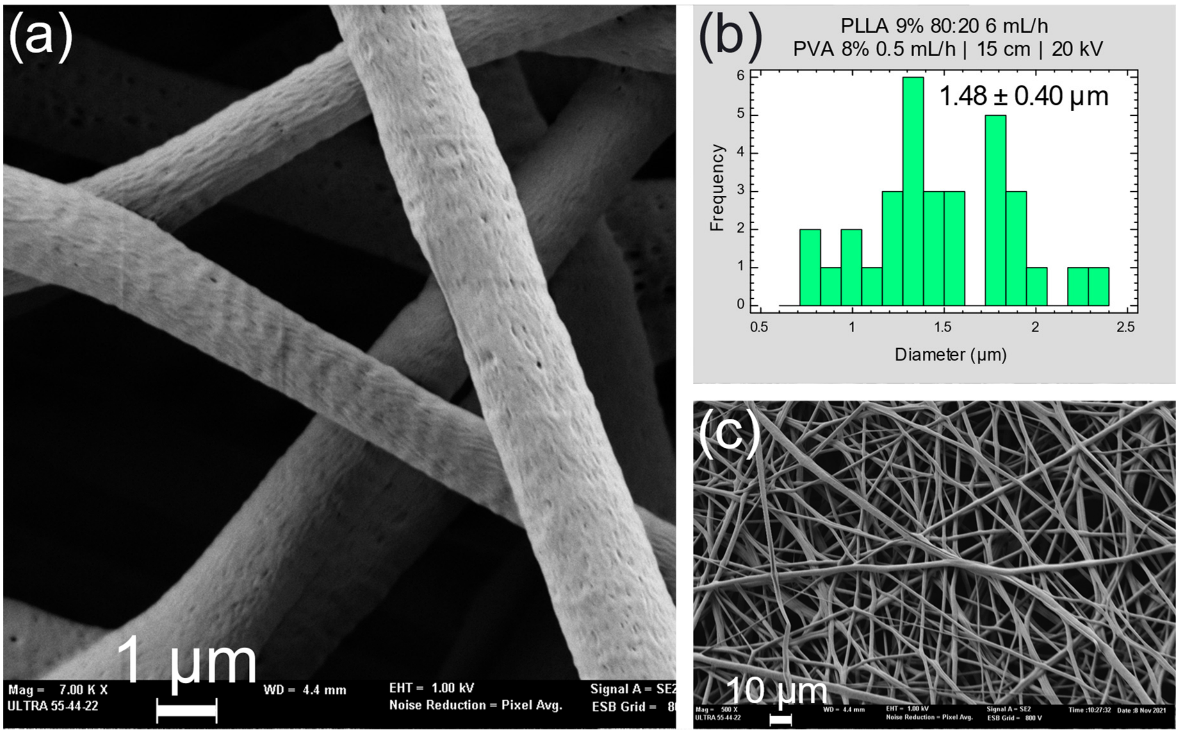

Figure S22. Coaxial microfibers of PLLA 9% chloroform:DMF 80:20 shell and PVA 8% core manufactured with shell – core flow 6 – 0.5 mL/h at 15 cm and 20 kV by FESEM (a,b) and histogram diameter results (mean ± SD = 1.48 ± 0.40 µm; range from 0.774 to 2.3 for) (b).

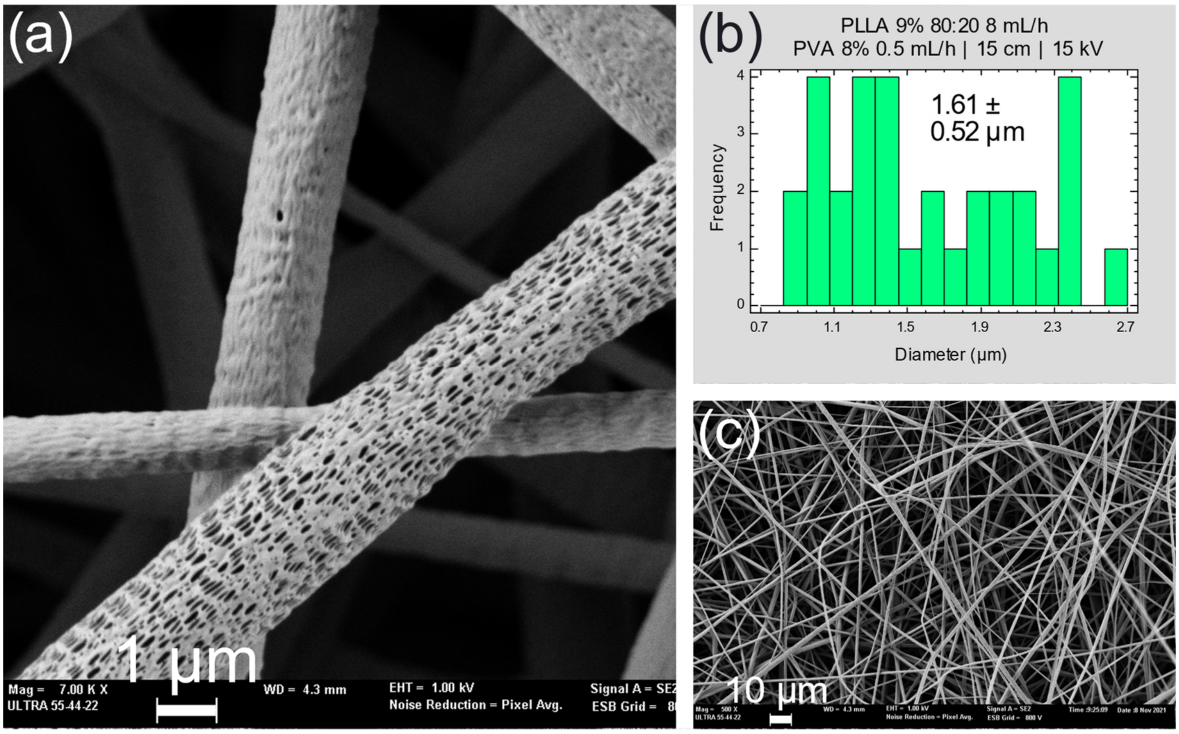

Figure S23. Coaxial microfibers of PLLA 9% chloroform:DMF 80:20 shell and PVA 8% core manufactured with shell – core flow 8 – 0.5 mL/h at 15 cm and 15 kV by FESEM (a, c) and histogram diameter results (mean ± SD = 1.61 ± 0.52 µm; range from 0.83 to 2.585) (b).

C-ES microfibers of PLLA 9% 100:0 | PVA 8% as a function of flow rate and voltage

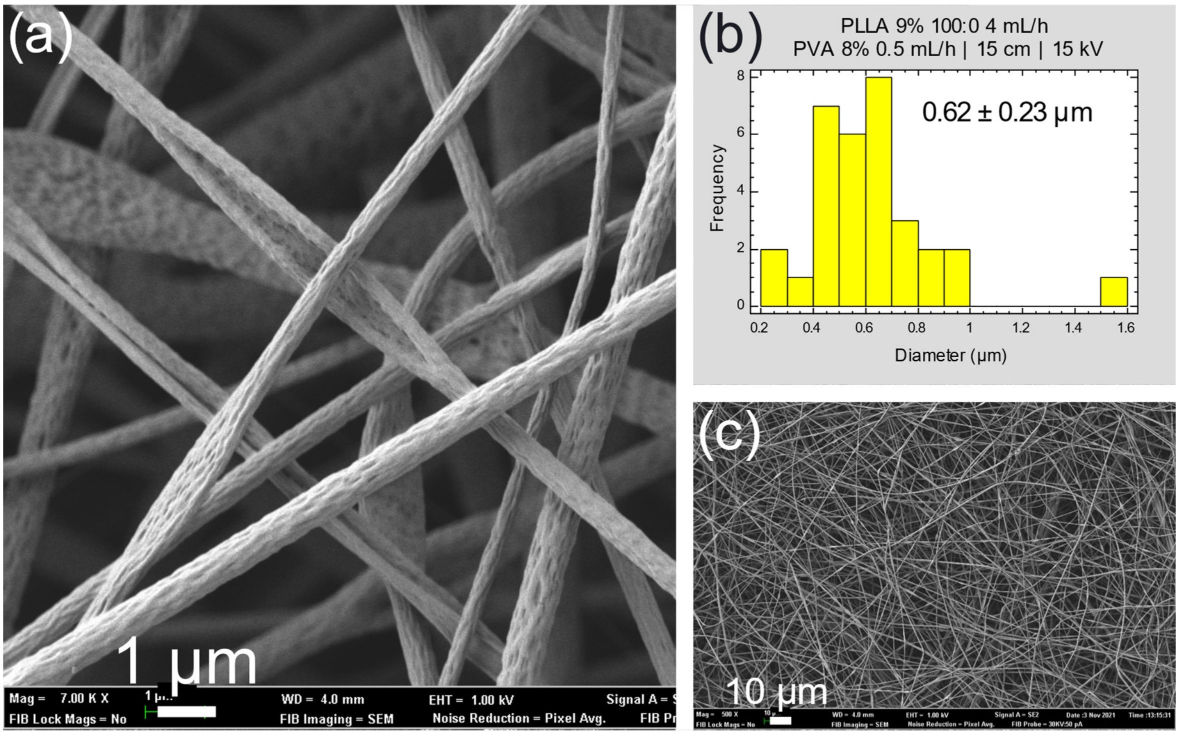

Figure S24. Coaxial microfibers of PLLA 9% chloroform:DMF 100:0 shell and PVA 8% core manufactured with shell – core flow 4 – 0.5 mL/h at 15 cm and 15 kV by FESEM (a, c) and histogram diameter results (mean ± SD = 0.62 ± 0.23 µm; range from 0.238 to 1.501) (b).

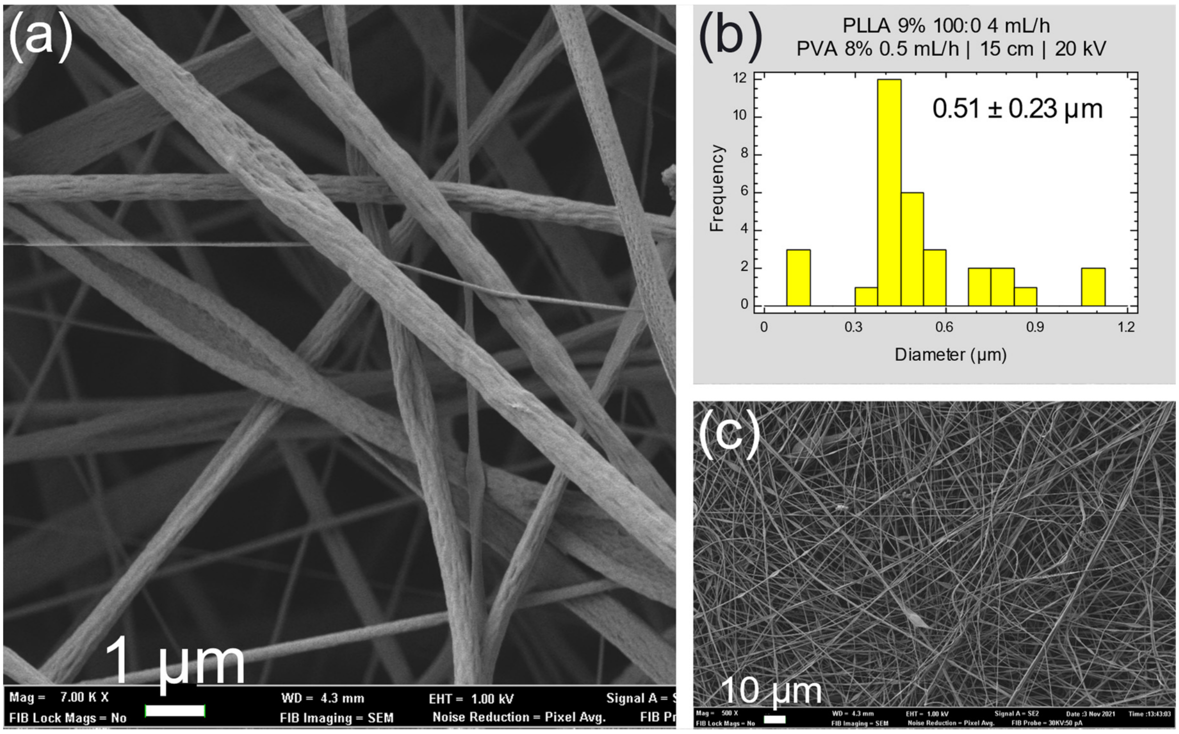

Figure S25. Coaxial microfibers of PLLA 9% chloroform:DMF 100:0 shell and PVA 8% core manufactured with shell – core flow 4 – 0.5 mL/h at 15 cm and 20 kV by FESEM (a, c) and histogram diameter results (mean ± SD = 0.51 ± 0.23 µm; range from 0.092 to 1.088) (b).

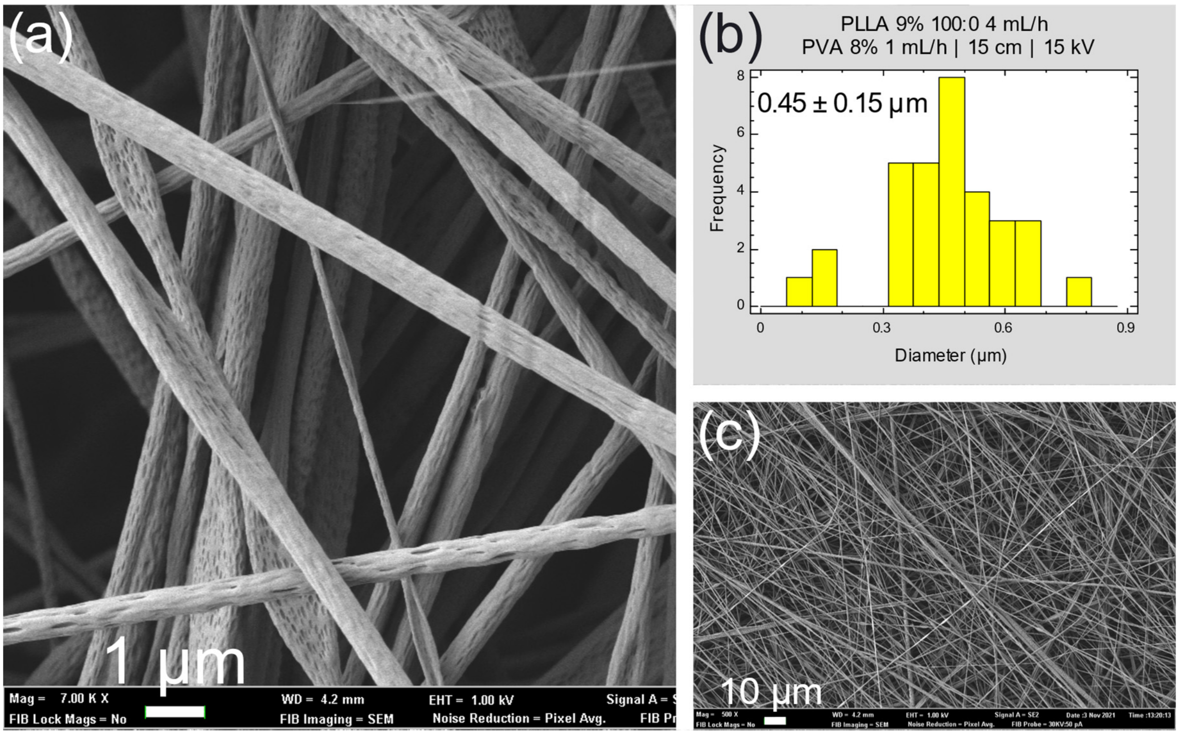

Figure S26. Coaxial microfibers of PLLA 9% chloroform:DMF 100:0 shell and PVA 8% core manufactured with shell – core flow 4 – 1 mL/h at 15 cm and 15 kV by FESEM (a, c) and histogram diameter results (mean ± SD = 0.45 ± 0.15 µm; range from 0.121 to 0.774) (b).

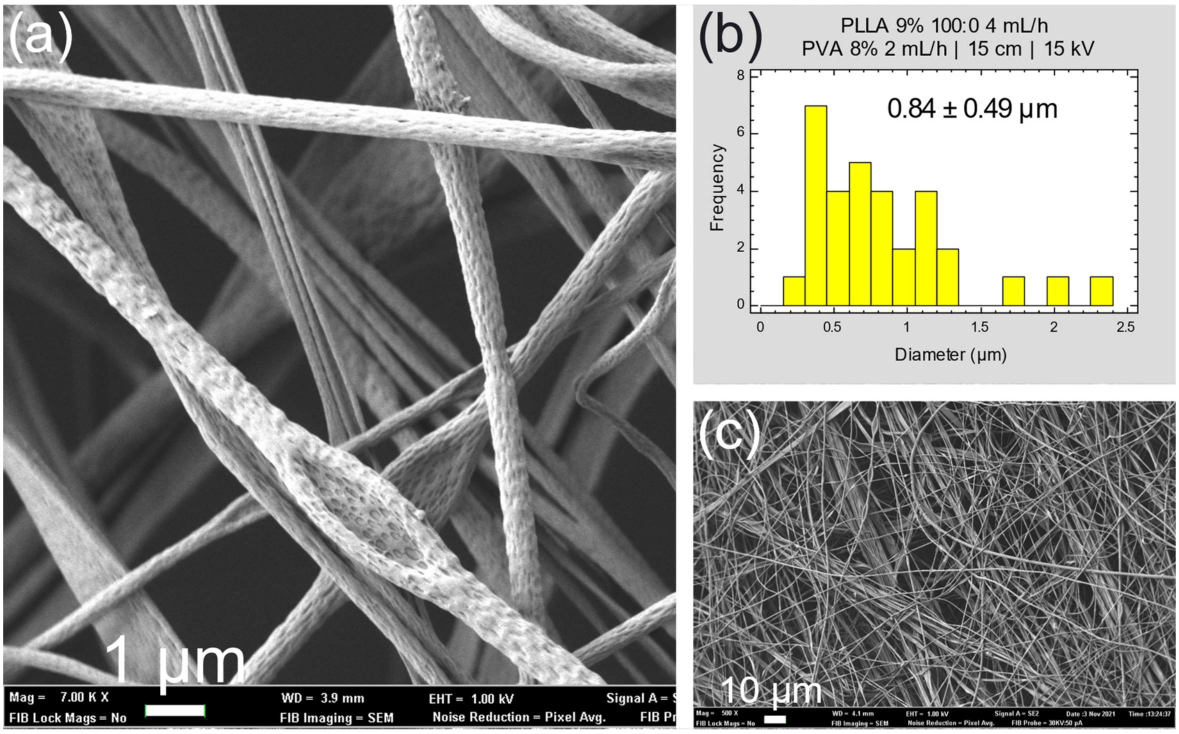

Figure S27. Coaxial microfibers of PLLA 9% chloroform:DMF 100:0 shell and PVA 8% core manufactured with shell – core flow 4 – 2 mL/h at 15 cm and 15 kV by FESEM (a, c) and histogram diameter results (mean ± SD = 0.84 ± 0.49 µm; range from 0.299 to 2.278) (b).

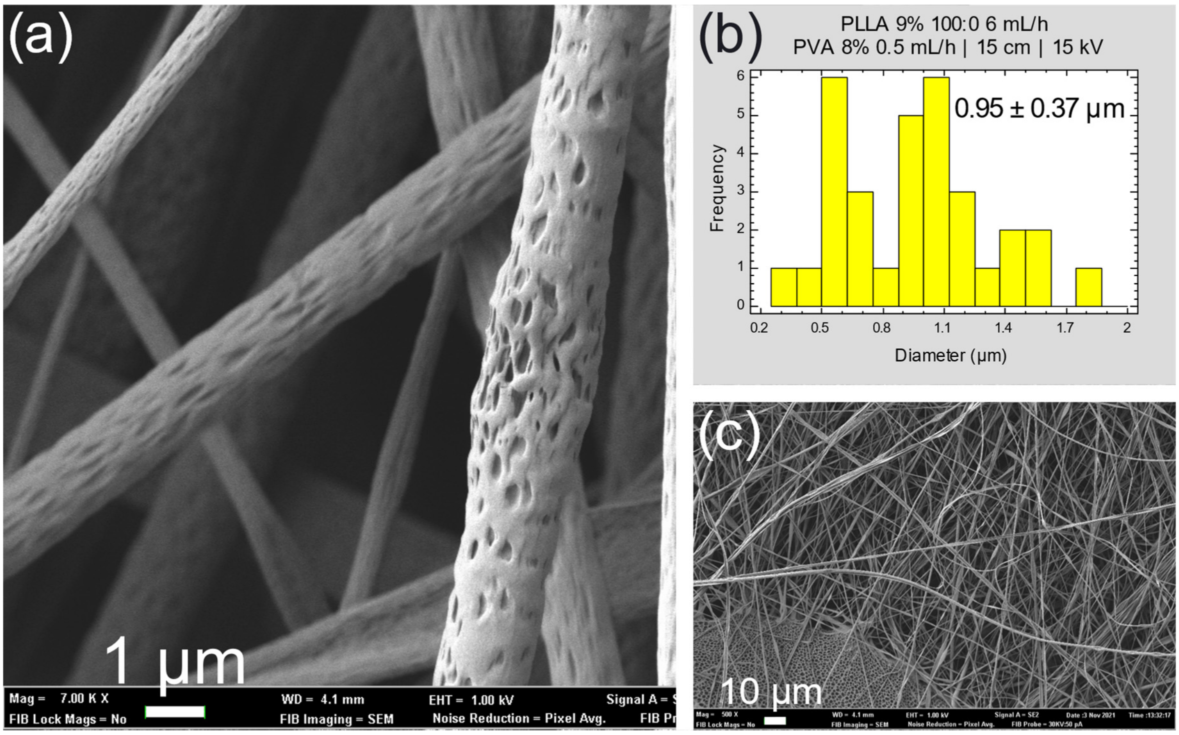

Figure S28. Coaxial microfibers of PLLA 9% chloroform:DMF 100:0 shell and PVA 8% core manufactured with shell – core flow 6 – 0.5 mL/h at 15 cm and 15 kV by FESEM (a, c) and histogram diameter results (mean ± SD = 0.95 ± 0.37 µm; range from 0.256 to 1.781) (b).

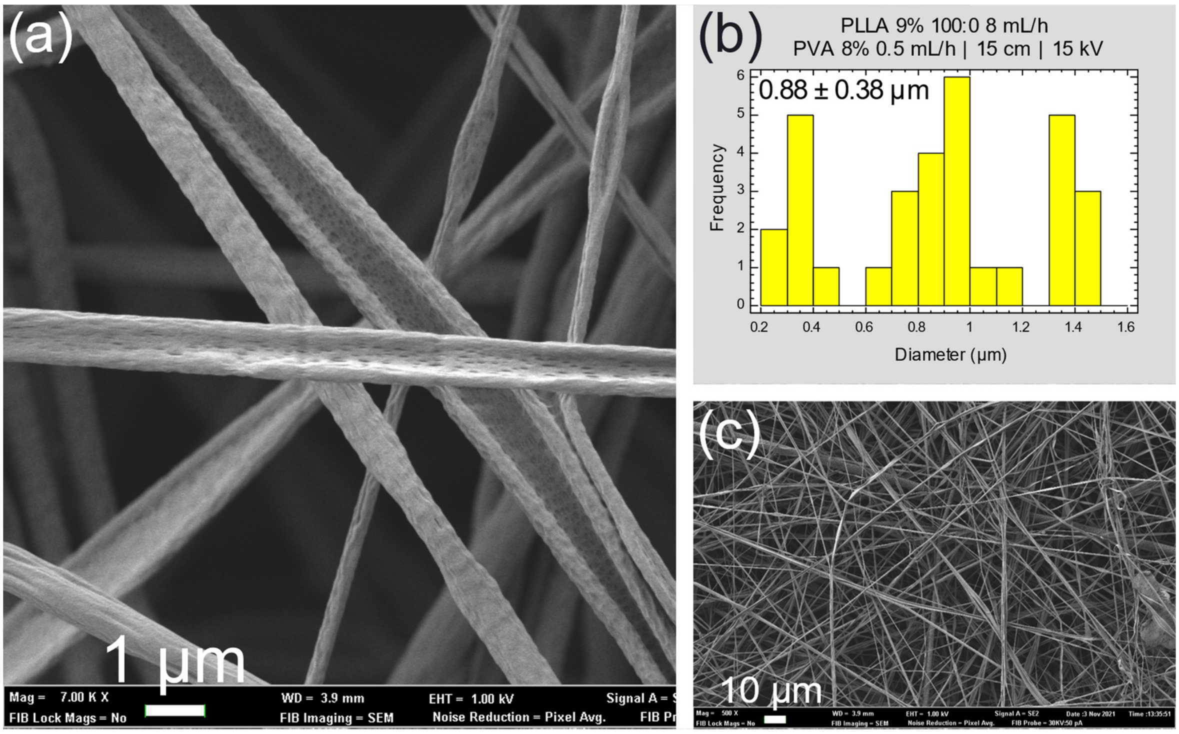

Figure S29. Coaxial microfibers of PLLA 9% chloroform:DMF 100:0 shell and PVA 8% core manufactured with shell – core flow 8 – 0.5 mL/h at 15 cm and 15 kV by FESEM (a, c) and histogram diameter results (mean ± SD = 0.88 ± 0.38 μm; range from 0.275 to 1.474) (b).
